# Integrating PET and MRI Radiomics for Staging and Prognostic Stratification in Anal Canal Cancer

**DOI:** 10.3390/cancers17223653

**Published:** 2025-11-14

**Authors:** Vanessa Murad, Andres Kohan, Lisa Avery, Claudia Ortega, Aruz Mesci, Patrick Veit-Haibach, Ur Metser

**Affiliations:** 1 Department of Medical Imaging, University of Toronto, Toronto, ON M5T 1W7, Canada; andreskohan1@gmail.com (A.K.); lisa.avery@uhn.ca (L.A.); claudia.ortega@uhn.ca (C.O.); patrick.veit-haibach@uhn.ca (P.V.-H.); ur.metser@uhn.ca (U.M.); 2 Princess Margaret Cancer Centre, Toronto, ON M5G 2C4, Canada; aruz.mesci@uhn.ca

**Keywords:** 18F-FDG PET/CT, magnetic resonance imaging, anal squamous cell carcinoma, radiomics

## Abstract

Introduction: While fluorine-18 fluorodeoxyglucose positron emission tomography combined with computed tomography (18F-FDG PET/CT = PET) and magnetic resonance imaging (MRI) is used for staging patients with anal squamous cell carcinoma (ASCC), the role of radiomics in predicting patients’ outcomes remains underexplored. This study aimed to assess whether PET- and MRI-derived radiomic features could improve stage discrimination and predict outcomes in ASCC. Methods: We retrospectively included 129 patients with biopsy-proven ASCC (2012–2023) staged with PET; 67 also underwent MRI at diagnosis. Both MRI and PET radiomic features were extracted from tumor volumes using LifeX. Cox proportional hazards models identified features predictive of progression-free survival (PFS). A two-feature prognostic model was developed using top-performing PET features. Features were also evaluated for their ability to distinguish early (I/II) from advanced (III/IV) stages according to the American Joint Committee on Cancer version 9 (AJCC v9). Time-dependent area under the curve (AUC) analyses assessed model performance over 1–5 years. Results: Twelve PET-derived radiomic features differentiated early-stage from advanced-stage tumors and predicted PFS, though none predicted overall survival (OS). A one-feature model based on tumor volume (SHAPE_Volume[log]) was significantly associated with PFS (HR: 1.85; *p* < 0.001), and a two-feature model incorporating gray-level zone length matrix—short-zone emphasis (GLZLM_SZE) improved long-term prediction (5-year AUC: 0.79 vs. 0.72). Twenty MRI features significantly discriminated between stage groups, but none were independently prognostic for survival. Notably, GLZLM_SZE showed significant differences across stage groups in both modalities, highlighting its potential as a cross-modality biomarker. Conclusions: The study highlights the potential of PET and MRI radiomics to aid staging and prognostication in ASCC patients. PET radiomic features are associated with survival outcomes, while PET and MRI features are associated with tumor stage.

## Simple Summary

This study examined whether radiomic features extracted from PET and MRI can enhance prognostication and staging in anal squamous cell carcinoma. A PET-derived shape feature, SHAPE_Volume(vx)(log), was a strong predictor of progression-free survival, and a two-feature model incorporating a heterogeneity measure (GLZLM_SZE) further improved long-term predictive performance. Additionally, eleven PET- and MRI-derived features reliably distinguished early (stage I/II) from advanced (stage III/IV) disease. These findings support the potential of integrated PET/MRI radiomics as non-invasive biomarkers for refined staging and individualized risk stratification, offering clinically meaningful information that may assist treatment planning and patient management.

## 1. Introduction

Anal canal cancer accounts for approximately 0.5% of new cancers and 0.3% of cancer-related deaths annually in the United States [1]. Its incidence is increasing globally, driven by a rise in human papillomavirus infection [2]. Anal squamous cell carcinoma (ASCC) is the most common subtype, constituting approximately 75% of cases [3]. The five-year survival rate for localized ASCC is around 82%, but this rate drops significantly in cases of locally advanced (66%) or metastatic disease (35%), with inguinal lymphadenopathy serving as an independent prognostic factor [4,5]. Imaging assessment is key for adequate staging, allowing for precise characterization of tumor extent, as well as locoregional and distant spread, which are critical for guiding treatment strategies [6,7]. The standard treatment for locally advanced anal squamous cell carcinoma consists of definitive chemoradiotherapy.

Magnetic resonance imaging (MRI) is a key tool for locoregional staging given its high spatial and soft tissue resolution [7]. Contrast-enhanced computed tomography (CT) of the chest, abdomen, and pelvis is recommended for screening distant metastases [8], and 18F-FDG PET/CT (=PET) is mainly considered for baseline staging in patients with confirmed nodal metastasis, or in patients with locally invasive tumors (cT3 or cT4) and no definitive nodal metastases on CT [9,10]. However, there is increasing evidence of the significant role of PET in staging and treatment decision-making for ASCC. For instance, Albertsson et al. [11] found that PET altered treatment plans in 25% of cases, and Jones et al. [12] described that PET upstaged nodal disease in 21% of patients and altered TNM (tumor, node, and metastasis) stage in 41% of patients. Furthermore, integrating MRI and PET for staging and therapy response assessment in ASCC has been shown to significantly improve diagnostic accuracy. A recent study by Adusumilli et al. [13] reported that combining both modalities for post-chemoradiotherapy (CRT) response evaluation yielded a positive predictive value of 78.9% compared to 36.7% for PET alone and 44.8% for MRI alone. These findings highlight the complementary value of PET and MRI in clinical practice.

Radiomics represents an advanced image analysis technique that extracts quantitative data from imaging (PET, CT, and MRI mainly), enabling detailed analysis of tissue characteristics like shape, heterogeneity, and texture, based on mathematical models [14,15]. Radiomic features have shown prognostic value across various cancers [16,17], aiding in predicting survival, treatment response, and recurrence risk. However, there is limited evidence of the role of PET and MRI radiomics in ASCC. A systematic review by Temperley et al. [18] identified nine studies regarding radiomics in ASCC, including a total of 589 patients, which addressed recurrence, progression-free survival (PFS), and prediction of human papillomavirus (HPV) status. They found radiomics to be promising for better risk stratification and personalized treatment planning in ASCC. The most significant study by Giraud et al. [19], evaluating MRI radiomics prognostic value in patients with non-metastatic ASCC who underwent CRT, concluded that MRI-derived radiomics may provide prognostic information when combined with clinical factors, potentially improving risk stratification and selection of treatment strategies. The aim of the present study is to determine the correlation between PET radiomic features and MRI radiomic features, with disease stage and outcomes in patients with ASCC.

## 2. Materials and Methods

### 2.1. Study Sample

**Figure 1:**
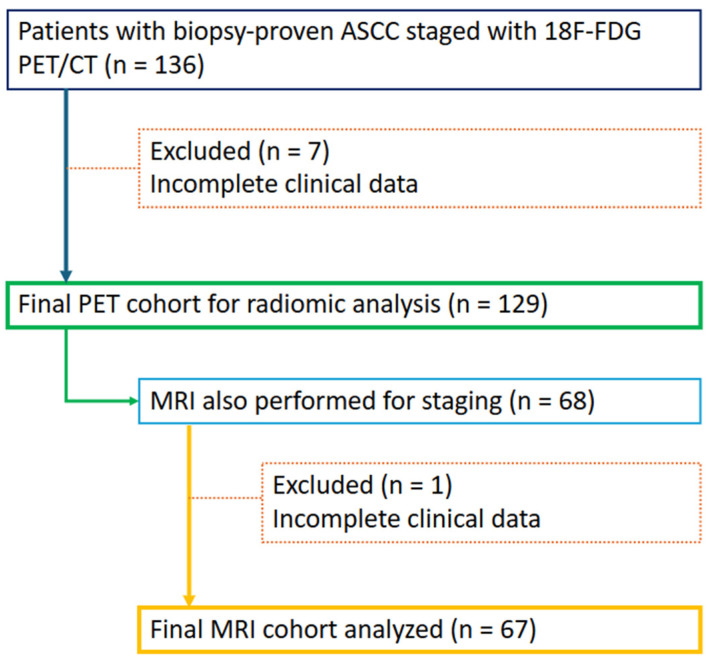
Flow diagram illustrating patient selection and inclusion for PET and MRI radiomic analyses.

Patients were retrospectively identified through the institutional Radiology Information System search engine and an existing provincial registry database of 18F-FDG PET/CT scans. A total of 129 patients aged ≥ 18 years with biopsy-confirmed ASCC who underwent 18F-FDG PET/CT for staging were included in this study, spanning an 11-year period from 1 January 2012 to 31 January 2023. Of these, 67 patients who had staging MRI at the same time were analyzed. Patients with incomplete datasets and with other tumor histologies (e.g., adenocarcinoma or adenosquamous carcinoma) were excluded (Figure 1). All patients underwent standard therapy consisting of CRT as per published clinical guidelines [20,21,22]. It should be noted that these patients are part of a larger cohort that was previously analyzed to assess the role of FDG PET/CT in the initial staging of ASCC [23].

### 2.2. Image Acquisition

**PET acquisition protocol**. 18F-FDG PET-CT images were acquired according to the institutional protocol from two different scanners: a Siemens mCT40 PET/CT (2010–2020) and a Siemens Vision scanner (2020–2023) (Siemens Healthineers, Knoxville, TN, USA). The mean injected dose of [18F] FDG was 384 MBq (range: 174–549), and the mean uptake time was 66 min (range: 50–122). Overall, the same parameters were used in both scanners: 5–9 bed positions were obtained, depending on patient height, with an acquisition time of 2–3 min per bed position. CT parameters were 120 kV, 3.0 mm slice width, 2.0 mm collimation, 0.8 sec rotation time, and 8.4 mm feed/rotation. A PET emission scan using time of flight with scatter correction was obtained covering the identical transverse field of view.

**MRI acquisition protocol.** MRI examinations were performed using either 1.5- or 3T systems (Avanto or Verio, Siemens Healthcare GmbH, Erlangen, Germany) with a phased-array coil. An intravenous antiperistaltic agent (20 mg of butylscopolamine bromide; Buscopan, Boehringer Ingelheim International GmbH, Ingelheim am Rhein, Germany) was used. The standard sequences included multiplanar T2-weighted fast spin-echo imaging, including high-resolution oblique imaging (perpendicular to the tumor axis in the sagittal view). In addition, the protocol included multiparametric MRI sequences, including diffusion-weighted and gadolinium contrast-enhanced MRI sequences. Parameters included a slice thickness of 3 mm, a field of view (FOV) of 16–22 cm, and an in-plane resolution of approximately 0.6–0.8 mm.

### 2.3. Data Extraction

**PET radiomics**: Tumors were contoured using LifeX v6.0. Initial tumor segmentation was performed using a semi-automatic SUVmax 2.5 threshold, consistent with prior oncologic PET radiomics studies, followed by manual review and correction by an expert radiologist with molecular imaging training (AK, >10 years of experience) to exclude physiologic or inflammatory uptake (e.g., sphincter, hemorrhoids, and bowel); When available, MRI was used for anatomic correlation to refine lesion boundaries. After the volume was approved, two other volumes were generated by reprocessing the original volume for 40% and 70% of the original threshold. Additional volumes at 40% and 70% SUVmax were generated to assess the stability of radiomic features across segmentation levels. Radiomics features were extracted for each of the three volumes generated.

**Figure 2:**
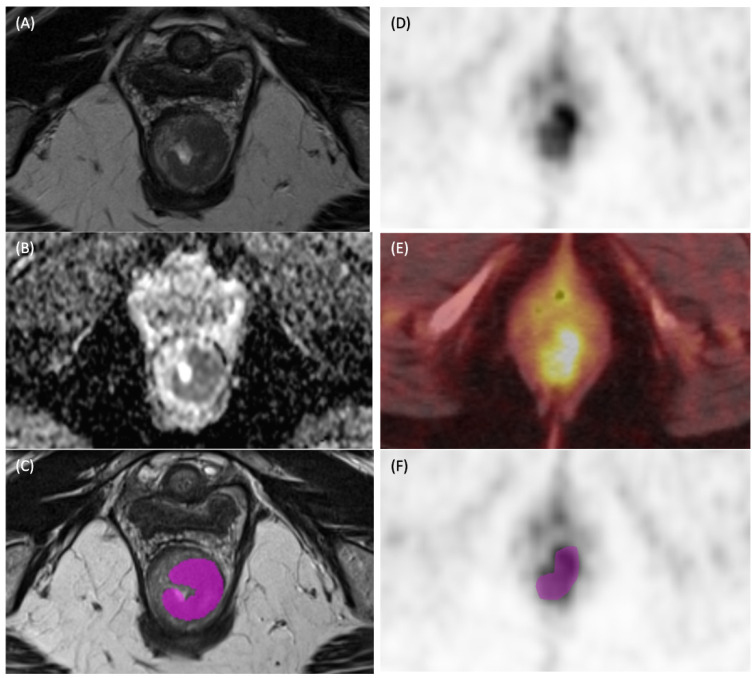
Representative images of tumor segmentation in a patient with anal squamous cell carcinoma. (**A**) Axial T2-weighted MRI image (T2WI) demonstrating the primary intermediate T2 signal intensity tumor with almost circumferential involvement of the anal canal. (**B**) Corresponding ADC map. (**C**) Axial T2WI showing corresponding manually contoured region of interest (ROI). (**D**) 18F-FDG PET Maximal intensity projection (MIP) image showing corresponding increased metabolic activity in the tumor. (**E**) Fused PET/CT image. (**F**) Corresponding manually contoured region of interest.

**MRI radiomics:** This was performed by a radiologist with 8 years of experience (VM). Mean Apparent Diffusion Coefficient (ADC) values were measured using the single-section method [24]. MRI radiomic features were extracted using LifeX v6.0. Tumor segmentation was performed primarily on high-resolution oblique T2-weighted images, with confirmation across perpendicular sagittal views to ensure complete and accurate delineation [25]. Parameterization involved meticulous configuration to ensure optimal settings, including the specification of bin widths for histogram features and distances for texture analysis, as recommended by the software developer [26] (Figure 2).

**Data analysis:** Sample characteristics were described with median and range for continuous variables and frequencies and percentages for categorical variables. PFS was calculated from the time of diagnosis to progression or death or censored at the date of the last follow-up. Similarly, overall survival was calculated from the time of diagnosis to death or censored at the last follow-up.

**PET radiomics**: To explore which radiomic features were associated with clinical outcome, the radiomic data were combined across the three imaging thresholds (2.5 cutoff, 40%, and 70%), and any features with no variability were removed. All features were screened for association with survival using the log-rank test, splitting the features at the median value. The mean significance of each feature across thresholds was calculated, features were ranked by this metric, and those with (unadjusted) values of *p* < 0.05 were selected for further analysis, known henceforth as the screened features. An examination of the screened features indicated that the 40% threshold data best discriminated survival, and so this threshold was chosen for all subsequent analyses. We examined distributions of the screened features and applied log transformations to skewed features to normalize them prior to Cox modeling. Univariate Cox proportional hazards models [27] were fit for each of the screened features, and *p*-values were adjusted to control for multiple testing using Holm’s correction. Starting with the first-ranked feature, a multivariable Cox model was created by selecting a second feature with the lowest absolute correlation with the first feature, from among the screened features. In this exploratory work, model building was stopped at two features to minimize the risk of overfitting. Tests of the proportional hazards (PH) assumption [28] were performed with a formal test of the null hypothesis of zero slope on the model parameters. Calibration of the one-feature and two-feature models was assessed using time-dependent AUC metrics at years one to five, with individual weighting to control for censoring as described by Li, Greene, and Hu (2018) [29]. Using the tdROC package version 1.0 in R software version 4.2.2 (R Foundation for Statistical Computing, Vienna, Austria) [30], 95% confidence intervals (CIs) were obtained using 100 bootstrap estimates. Kaplan–Meier curves were used to visualize the discriminative ability of the models by dividing the sample into quartiles based on the model risk score. Features with the ability to discriminate high-level AJCCv9 staging (levels I/II vs. III/IV) were identified with Wilcoxon rank sum tests, selecting features statistically significant (*p* < 0.05) after applying Holm’s correction.

**MRI radiomics**: Similar processes were followed for the radiomic data obtained from MRI, without the threshold selection step. All statistical analyses were conducted using the R statistical programming language version 4.4.2 [31].

## 3. Results

A total of 129 patients who underwent 18F-FDG PET/CT for staging and 67 patients who underwent MRI were analyzed. General characteristics of both groups are detailed in Table 1.

**PET observations:** In total, 12 of 191 (6.3%) radiomic features discriminated between early-stage versus advanced-stage disease (AJCCv9 stages I/II versus III/IV) and were also predictive of PFS; these are shown in Table 2. No features were significant predictors of OS. Expanded Cox models results and tests for differences between stages are reported in Supplementary Tables S1 and S2, respectively.

None of the PET-derived radiomic features were significant predictors of OS. An initial univariate Cox model identified a shape-based radiomic feature—SHAPE_Volume(vx)(log)—as a strong predictor of PFS, and this feature was used to construct a one-feature model. To improve prognostic performance, a two-feature Cox model incorporating both SHAPE_Volume(vx)(log) and GLZLM_SZE was also developed; GLZLM_SZE was selected as the second feature because it exhibited the lowest correlation with SHAPE_Volume(vx)(log) among the screened variables, providing complementary information on intratumoral heterogeneity. This combination enhanced model robustness and interpretability by integrating volumetric and textural characteristics. In the one-feature model, SHAPE_Volume(vx)(log) showed a hazard ratio (HR) of 1.85 (95% CI: 1.45–2.36; *p* < 0.001). In the two-feature model, SHAPE_Volume(vx)(log) remained significant with an HR of 1.85 (95% CI: 1.47–2.33; *p* < 0.001), and GLZLM_SZE was inversely associated with risk, with an HR of 0.05 (95% CI: 0.0061–0.45; *p* = 0.007). Both features remained statistically significant after Holm’s correction for multiple testing, suggesting a robust association with progression-free survival.

**Figure 3:**
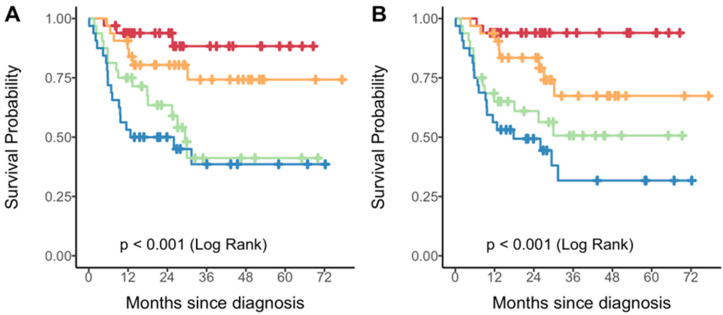
Kaplan–Meier curves of progression-free survival by quartiles of the radiomic score derived from a single feature (**A**) or two features (**B**). Each color represents a specific quartile of the radiomic score: Q1 = red; Q2 = orange; Q3 = green; Q4 = blue.

**Figure 4:**
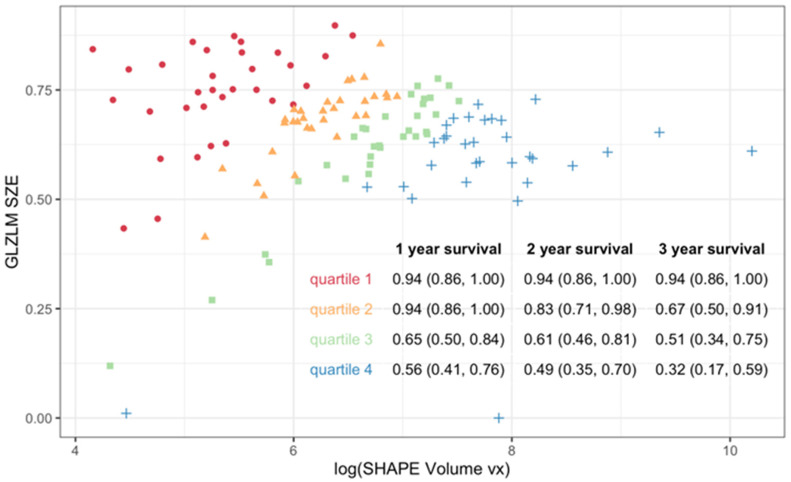
Radiomic values and progression-free survival probabilities for the PET-derived two-feature model. Scatter plot of SHAPE_Volume(vx)(log) versus GLZLM_SZE values with colors representing quartiles of the radiomic risk score (Q1 = red; Q2 = orange; Q3 = green; Q4 = blue). The table insert summarizes progression-free survival probabilities for each quartile at different time intervals. Density plots show the distribution of radiomic scores across quartiles, with clear clustering patterns.

A radiomic score was derived from each model and used to stratify the PET sample into quartiles. Kaplan–Meier analysis demonstrated discrimination in PFS across score quartiles for both models (Figure 3), with better discrimination in the two-feature model (log-rank *p* < 0.001). Patients in the highest quartile of the radiomic score consistently showed worse outcomes compared to those in the lowest quartile. As shown in Figure 4, the radiomic score quartile revealed clear clustering patterns. Time-dependent AUC values for predicting PFS at 1 to 5 years are shown in Table 3. The two-feature model consistently outperformed the one-feature model, with the greatest improvements observed at later timepoints.

**MRI observations:** After controlling for multiple testing, no MRI features were associated with PFS. Table 4 lists the MRI features that significantly discriminated between early- and advanced-stage disease.

**Figure 5:**
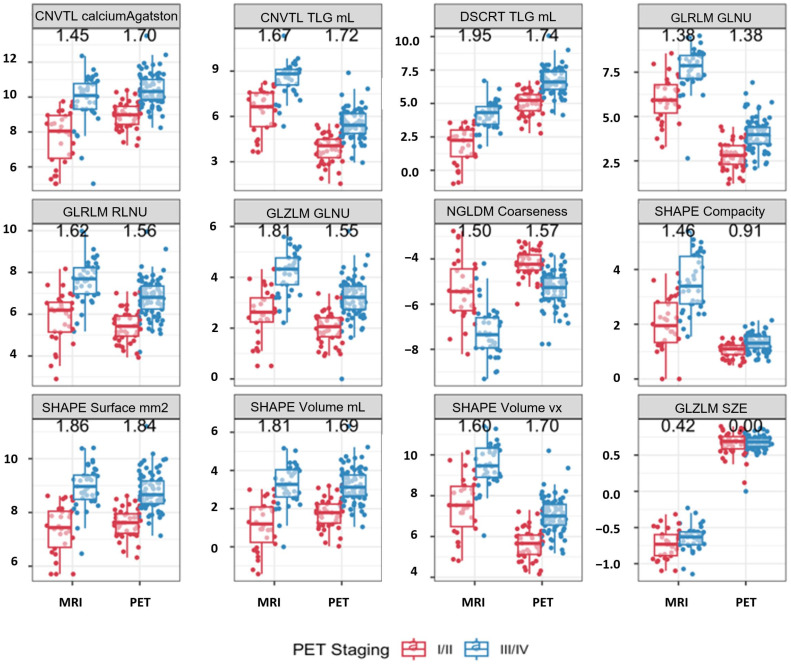
Comparison of radiomic feature values between early-stage (AJCC v9 stage I/II, red) and advanced-stage (AJCC v9 stage III/IV, blue) anal squamous cell carcinoma, measured on both MRI and PET for each feature. The numbers shown above each panel correspond to the Cohen’s *d* effect sizes for MRI (left) and PET (right), indicating the magnitude of difference between early- and advanced-stage groups. Larger absolute values denote stronger discrimination between staging groups. CNVTL = conventional; DSCRT = discretized; GLRLM = gray-level run length matrix; GLNU = gray-level non-uniformity; RLNU = run length non-uniformity; NGLDM = neighborhood gray-level difference matrix; GLZLM = gray-level zone length matrix; SZE = short-zone emphasis; TLG = total lesion glycolysis; SHAPE = shape feature.

**Figure 6:**
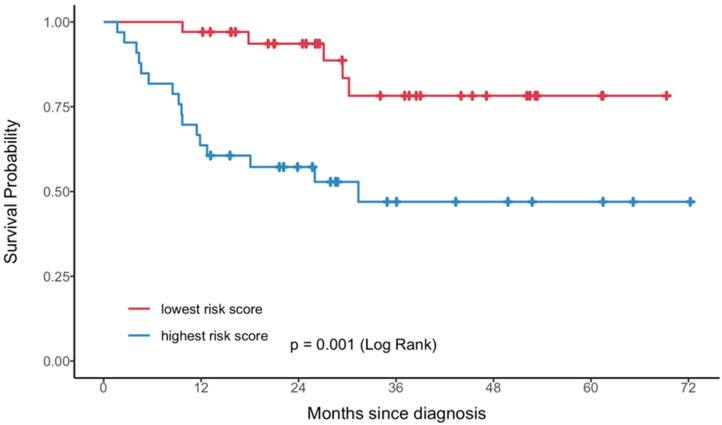
Kaplan–Meier curves illustrating progression-free survival based on MRI-derived radiomic risk scores. Patients were stratified into groups with the lowest (red) and highest (blue) radiomic risk scores derived from the multivariable Cox model. A significantly shorter progression-free survival was observed in the high-risk group (log-rank *p* = 0.001).

**Figure 7:**
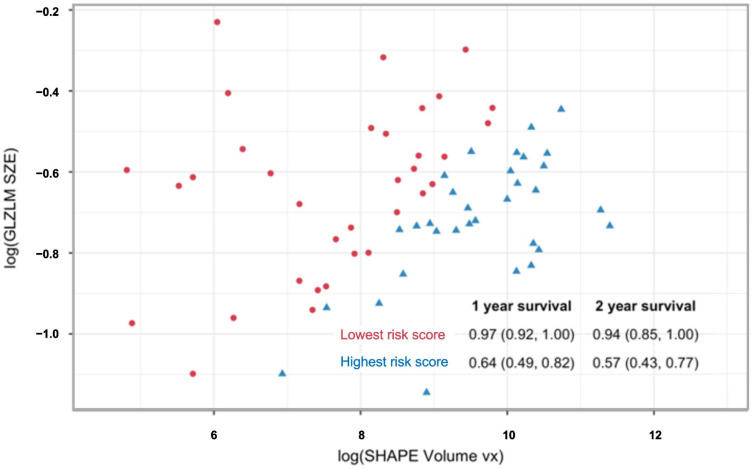
Scatter plot illustrating the relationship between the two MRI-derived radiomic features—SHAPE_Volume(vx) and GLZLM_SZE—used in the multivariable Cox model for progression-free survival. Each point represents an individual tumor, color-coded according to radiomic risk group (red = lowest risk score; blue = highest risk score). The inset table summarizes 1-, and 2-year progression-free survival probabilities (95% confidence intervals) for each risk group, demonstrating the prognostic discrimination achieved by the model.

**Integrating PET and PET features:** Eleven radiomic features were found to significantly differentiate tumor stage (AJCC v9 I/II vs. III/IV) across both PET and MRI. Figure 5 illustrates the feature values across groups for both imaging modalities, as well as GLZLM_SZE, which contributed to the PET model of survival. Effect size differences, measured using Cohen’s *d*, were used to assess whether MRI or PET provided stronger discrimination between staging groups. Overall, PET features demonstrated slightly larger effect sizes than MRI for most parameters, indicating stronger separation between early and advanced stages. Features such as SHAPE_Volume, SHAPE_Surface, GLRLM_GLNU, and GLZLM_GLNU showed the highest effect sizes (up to *d* = 1.8), reflecting marked differences in tumor morphology and heterogeneity between stage groups. MRI-derived features followed similar trends but with generally smaller magnitudes. GLZLM_SZE, which contributed to the PET survival model, exhibited consistent differences in both PET and MRI datasets, suggesting its potential as a cross-modality biomarker. Although our small MRI sample did not indicate any significant predictors of survival after controlling for multiplicity, we fit our multivariable Cox model on the MRI data, finding that SHAPE Volume and GLZLM SZE, when measured with MRI, were predictive of PFS (Figure 6). Figure 7 illustrates the relationship between MRI-derived SHAPE Volume and GLZLM SZE, highlighting the distinction between high- and low-risk groups.

## 4. Discussion

While significant advances have been made in the staging and management of ASCC, the integration of advanced imaging modalities like PET and MRI radiomics remains an area of investigation. This study examined the correlation between PET and MRI radiomic features with staging and clinical outcomes. Twelve PET-based radiomic features significantly discriminated between early- and advanced-stage disease (AJCC v9 stage I/II versus III/IV). These features, reflecting tumor biology such as shape complexity and intratumoral heterogeneity, may aid in improving risk stratification and treatment planning [32,33]. These features could also play a crucial role in enhancing clinical staging accuracy, particularly in cases where staging is equivocal, potentially reducing misclassification and improving outcomes. Furthermore, identifying patients at high risk for recurrence may justify intensifying post-therapy surveillance strategies.

None of the PET-derived radiomic features were significant predictors of OS in our study; however, time-dependent AUC analysis demonstrated the predictive value of those same 12 PET radiomic features for PFS. Although several studies have demonstrated the predictive value of various PET-derived radiomic features, the results remain inconsistent. For instance, Brown et al. [34] identified GLCM entropy and NGLDM busyness as the most predictive features, whereas Wang et al. [35] highlighted roundness. We created one-feature and two-feature models to predict PFS. The one-feature, shape-based model [SHAPE_Volume(vx)(log)] demonstrated a significant association with risk of progression (HR: 1.85; 95% CI: 1.45–2.36; *p* < 0.001). The two-feature model incorporating a second heterogeneity-based feature (GLZLM_SZE) showed improved discrimination, with GLZLM_SZE inversely associated with risk (HR: 0.05; 95% CI: 0.0061–0.45; *p* = 0.007). Stratifying the sample by quartiles showed that those tumors with both high volume and high heterogeneity features were associated with the poorest outcomes, whereas small-volume tumors showed favorable survival regardless of heterogeneity. Time-dependent AUC analysis revealed comparable short-term predictive performance between models, but superior long-term prediction with the two-feature model, particularly from year three onward (AUC: 0.79 vs. 0.72 at five years). These findings highlight the potential for radiomics to enhance tumor staging and treatment decision-making. Future research should focus on validating features that have previously shown significant correlations with strong predictive performance, which could be prioritized in future models to improve reproducibility.

We identified 20 out of 97 MRI-derived radiomic features able to discriminate between patients with stage III/IV and stage I/II disease, supporting its role in initial diagnosis. These included both shape-based features (e.g., SHAPE_Volume, SHAPE_Sphericity, and SHAPE_Surface) and texture-based features (e.g., GLRLM, GLZLM, and NGLDM matrices), suggesting that MRI radiomics may potentially reflect tumor morphology and heterogeneity. Integrating radiomic features with clinical information at the time of initial diagnosis could add value to risk stratification and potentially impact management.

While our study did not demonstrate that MRI-derived features, such as ADC values or MRI radiomic features, are independently prognostic, prior research suggests that integrating MRI radiomics with clinicopathological models may provide significant prognostic value. Giraud et al. [19] developed a pre-therapeutic MRI radiomic model and identified at least two features (FirstOrder_Entropy and GLCM_JointEnergy) that, when combined with clinical parameters, provided prognostic information. Similarly, Owczarczyk et al. [36] explored the predictive potential of pre- and post-treatment MRI texture features extracted from T2-weighted and diffusion-weighted imaging, finding that integrating these features with clinicopathological data improved risk reclassification. Previous work has explored the prognostic value of pre- and post-CRT MRI texture feature values, showing that tumors with both high volume and high heterogeneity were associated with the poorest outcomes, whereas small-volume tumors showed favorable survival regardless of heterogeneity. Finally, Jones et al. [12,37] identified strong associations between ADC metrics from DWI and local recurrence of ASCC, also supporting the prognostic value of MRI-derived features in this disease.

Moreover, integrating radiomic features from PET and MRI could enhance both staging and outcome prediction in patients with ASCC. Eleven radiomic features exhibited significant differentiation of tumor stage across both modalities, suggesting that their complementary strengths could further refine accuracy. For instance, features such as SHAPE Volume, SHAPE Compacity, and GLRLM GLNU showed similar trends across both modalities. Furthermore, GLZLM_SZE, which contributed to the PET survival model, exhibited consistent behavior across PET and MRI datasets, demonstrating an inverse association with progression risk and measurable differences between stage groups. Although its statistical significance varies between modalities, this reproducible pattern supports its potential as a cross-modality texture descriptor. Such features may serve as the foundation for integrated biomarker models aimed at optimizing patient selection for treatment and enhancing personalized therapeutic approaches. Of note, radiomic features such as SHAPE_Volume and GLZLM_SZE have demonstrated prognostic relevance in other malignancies such as head and neck, lung, and rectal cancers, supporting their potential as generalizable imaging biomarkers [26,32].

This study has several limitations. First, its retrospective nature may introduce selection bias and limit the generalizability of findings. Second, variability in MRI protocols and radiomic feature extraction from both PET and MRI remains a challenge. Although acquisition parameters were standardized across scanners from the same manufacturer, minor differences in hardware and reconstruction may still influence radiomic features, particularly in PET imaging. Future studies should incorporate harmonization techniques to improve reproducibility and enable multi-institutional validation. Given the potential impact of small lesion volumes on texture stability, all features were extracted using strict LIFEx parameters to ensure adequate voxel representation; Features demonstrating instability or low variability across thresholds were excluded from further analysis, reducing bias associated with small-volume effects. Other potential confounding factors, such as comorbidities and inter-patient variability, may also impact radiomic features. Additionally, tumor heterogeneity and variations in the tumor microenvironment may influence radiomic patterns, complicating interpretation and reproducibility. Given the study’s modest sample size and limited number of progression events, a separate validation set could not be retained. To minimize overfitting and enhance robustness, we employed multiple methodological safeguards, including non-parametric log-rank testing for initial screening, Cox proportional hazards modeling with Holm’s correction for multiple comparisons, and bootstrap resampling (100 iterations) for internal validation. Future multicenter studies with larger cohorts should incorporate full cross-validation to further confirm model stability. A key strength of this study is the cross-validation of radiomic features in the MRI sub-sample, demonstrating that the model is robust across different imaging modalities and regions of interest. Future studies with larger, prospective cohorts are needed to refine and validate these findings.

## 5. Conclusions

This study highlights the potential of PET and MRI radiomics in improving staging and prognostication in patients with ASCC. PET-derived radiomic features showed strong associations with both disease stage and PFS, while MRI-derived features aid in stage categorization, particularly those associated with tumor morphology and heterogeneity. PET-derived feature models effectively predicted PFS with strong long-term performance, potentially providing relevant information for risk assessment. Given the complementary nature of PET and MRI, integrating radiomic features from both modalities could enhance risk stratification and clinical decision-making. Future prospective trials should validate these findings and propose cross-modality biomarker models integrating clinicopathological features to further refine personalized treatment strategies in ASCC.

## Figures and Tables

**Table 1:** Characteristics of study participants. Values presented are n (%) for categorical variables and median (range) for continuous variables.

Covariate	Full Sample (n = 129)	MRI Available (n = 67)
Gender		
Female	67 (52)	31 (46)
Male	62 (48)	36 (54)
Age at Dx		
Mean (sd)	61.7 (11.5)	60.6 (11.3)
Median (Min, Max)	61 (33, 92)	60 (33, 87)
Primary Tumor Location		
Anal Canal	129 (100)	67 (100)
Histology		
Squamous Cell Carcinoma	129 (100)	67 (100)
Differentiation		
Moderate	29 (26)	12 (20)
Poor	12 (11)	7 (12)
Unknown	57 (50)	38 (63)
Well	15 (13)	3 (5)
Missing	16	7
LVSI Status		
Negative	3 (2)	1 (1)
Positive	2 (2)	
Unknown	124 (96)	66 (99)
TNM Staging		
T Stage (Based on MRI)		
T1		5 (7)
T2		31 (46)
T3		19 (28)
T4		12 (18)
N Stage (Based on MRI and PET/CT)		
N0		26 (39)
N1a		24 (36)
N1c		17 (25)
M Stage (Based on MRI and PET/CT)		
M0		60 (90)
M1		7 (10)
PET Stage AJCC 9th Edition		
I	3 (2)	2 (3)
IIA	18 (15)	15 (22)
IIB	23 (19)	13 (19)
IIIA	48 (39)	26 (39)
IIIB	1 (1)	1 (1)
IIIC	6 (5)	1 (1)
IV	25 (20)	9 (13)
Missing	5	
Follow-Up Months		
Mean (sd)	28.7 (18.4)	32.2 (17.1)
Median (Min, Max)	25.7 (1.7, 77.4)	27.9 (1.7, 72.2)

**Table 2:** Distributions (median and IQR) of PET radiomic features for those in stage I/II vs. stage III/IV, and hazard ratios from Cox proportional hazards models for progression-free survival. Levels of significance were adjusted (in each analysis) for multiple comparisons using Holm’s correction. Only radiomic features that were statistically significant under both analyses are shown.

Covariate	I/II (n = 44)	III/IV (n = 79)	Stage Difference *p*-Value	HR (95%CI)	Cox Model *p*-Value
CONVENTIONAL SUVbwcalciumAgatstonScore [onlyForCT] (log)	9.0 (8.4–9.5)	10.3 (9.9–11.0)	<0.001	1.86 (1.46, 2.37)	<0.001
CONVENTIONAL TLG (mL) [onlyForPETorNM] (log)	4.1 (3.3–4.5)	5.4 (4.9–6.2)	<0.001	1.63 (1.29, 2.06)	<0.001
DISCRETIZED TLG (mL) [onlyForPETorNM] (log)	5.2 (4.5–5.7)	6.6 (6.1–7.4)	<0.001	1.63 (1.29, 2.05)	<0.001
SHAPE Volume (mL)(log)	1.8 (1.2–2.2)	3.1 (2.7–3.8)	<0.001	1.85 (1.46, 2.36)	<0.001
SHAPE Volume (vx)(log)	5.7 (5.1–6.1)	7.0 (6.6–7.6)	<0.001	1.85 (1.45, 2.36)	<0.001
SHAPE Surface (mm^2^) [onlyFor3DROI] (log)	7.6 (7.2–7.9)	8.7 (8.3–9.2)	<0.001	2.27 (1.68, 3.06)	<0.001
SHAPE Compacity [onlyFor3DROI] (log)	1.1 (0.9–1.2)	1.3 (1.1–1.6)	<0.001	4.44 (1.69, 11.71)	0.018
GLRLM GLNU (log)	2.8 (2.3–3.4)	4.0 (3.5–4.4)	<0.001	1.98 (1.53, 2.55)	<0.001
GLRLM RLNU (log)	5.4 (5.0–5.9)	6.8 (6.2–7.3)	<0.001	1.78 (1.39, 2.29)	<0.001
NGLDM Coarseness (log)	−4.2 (−4.5–(−3.8))	−5.3 (−5.7–(−4.8))	<0.001	0.51 (0.38, 0.69)	<0.001
NGLDM Busyness (log)	−1.8 (−2.4–(−1.2))	−1.2 (−1.6–(−0.7))	0.001	1.89 (1.34, 2.66)	0.002
GLZLM GLNU (log)	2.1 (1.7–2.4)	3.2 (2.7–3.7)	<0.001	2.02 (1.51, 2.70)	<0.001

SUV = standardized uptake value; TLG = total lesion glycolysis; ROI = region of interest; GLRLM = gray-level run length matrix; GLNU = gray-level non-uniformity; RLNU = run length non-uniformity; NGLDM = neighborhood gray-level difference matrix; GLZLM = gray-level zone length matrix; PH = proportional hazards; HR = hazard ratio; CI = confidence interval.

**Table 3:** Time-dependent area under the receiver operating curve for prediction of progression-free survival at various timepoints for the one-feature and two-feature models.

Year	One-Feature Model	Two-Feature Model
1	0.76 (0.69, 0.86)	0.77 (0.71, 0.87)
2	0.73 (0.66, 0.83)	0.75 (0.68, 0.85)
3	0.72 (0.61, 0.83)	0.79 (0.71, 0.87)
4	0.72 (0.61, 0.83)	0.79 (0.71, 0.87)
5	0.72 (0.61, 0.83)	0.79 (0.71, 0.87)

**Table 4:** MRI radiomic features that discriminate between patients with stage III/IV vs. stage I/II disease based on PET AJCC v9. Wilcoxon rank sum test was used to compare the difference in distributions between groups; Holm’s corrections were made to the *p*-values to control for multiple testing, and only features with adjusted *p* < 0.05 are shown.

Feature	I/II (n = 30)	III/IV (n = 37)	*p*-Value
Greater dimension cm	3.0 (2.4–3.8)	6.1 (4.5–7.3)	<0.001
SHAPE Compacity onlyFor3DROI	1.9 (1.3–2.8)	3.4 (2.7–4.5)	<0.001
CNVTL calciumAgatstonScore onlyForCT (log)	8.0 (6.5–8.9)	10.1 (9.3–10.8)	<0.001
CNVTL TLG mL onlyForPETorNM (log)	6.6 (5.3–7.6)	8.8 (8.1–9.1)	<0.001
DSCRT Kurtosis (log)	1.1 (0.8–1.4)	1.5 (1.4–1.7)	0.049
DSCRT ExcessKurtosis (log)	−4.0 (−4.0–0.1)	0.5 (−0.1–1.0)	0.044
DSCRT TLG mL onlyForPETorNM (log)	2.2 (1.0–3.0)	4.3 (3.4–4.8)	<0.001
SHAPE Volume mL (log)	1.2 (0.2–2.1)	3.3 (2.6–4.0)	<0.001
SHAPE Volume vx (log)	7.5 (6.5–8.5)	9.5 (8.9–10.3)	<0.001
SHAPE Sphericity onlyFor3DROI (log)	−0.5 (−0.5–(−0.4))	−0.6 (−0.7–(−0.5))	0.031
SHAPE Surface mm^2^ onlyFor3DROI (log)	7.4 (6.7–8.0)	9.0 (8.5–9.4)	<0.001
GLRLM LRHGE (log)	3.9 (3.1–4.5)	4.7 (4.3–5.0)	0.003
GLRLM GLNU (log)	5.9 (5.2–6.8)	7.9 (7.1–8.4)	<0.001
GLRLM RLNU (log)	6.2 (5.1–6.6)	7.7 (7.0–8.2)	<0.001
NGLDM Coarseness (log)	−5.4 (−6.3–(−4.4))	−7.4 (−7.9–(−6.6))	<0.001
NGLDM Contrast (log)	−4.0 (−4.5–(−3.5))	−4.7 (−5.1–(−4.5))	0.021
GLZLM LZE (log)	10.9 (9.7–12.2)	13.2 (12.2–14.1)	0.007
GLZLM LZHGE (log)	13.0 (10.8–13.7)	14.8 (14.0–15.7)	<0.001
GLZLM GLNU (log)	2.6 (2.2–3.2)	4.3 (3.7–4.8)	<0.001
GLZLM ZLNU (log)	2.2 (1.3–2.8)	4.0 (3.5–4.5)	<0.001

SHAPE = shape; CNVTL = conventional; DSCRT = discretized; GLRLM = gray-level run length matrix; GLNU = gray-level non-uniformity; RLNU = run length non-uniformity; LRHGE = long-run high gray-level emphasis; NGLDM = neighborhood gray-level difference matrix; GLZLM = gray-level zone length matrix; LZE = long-zone emphasis; LZHGE = long-zone high gray-level emphasis; ZLNU = zone length non-uniformity; TLG = total lesion glycolysis.

## Data Availability

The datasets generated and analyzed during the current study are not publicly available due to institutional and patient privacy regulations but are available from the corresponding author upon reasonable request and with appropriate research ethics approval.

## Supplementary Materials

The following supporting information can be downloaded at https://www.mdpi.com/article/10.3390/cancers17223653/s1, Supplementary Table S1: Univariate hazard ratios associated with progression from separate Cox PH models. Note that the HR are dependent on the distribution of the features and not readily interpretable; Supplementary Table S2: PET radiomic features that discriminate between patients with stage III/IV vs. stage I/II disease based on PET AJCC v9. Wilcoxon rank sum test was used to compare the difference in distributions between groups, Holm’s corrections were made to the *p*-values to control for multiple comparisons and features with adjusted *p*-values < 0.05 are shown.
