# Supplementary material for: Integrating PET and MRI Radiomics for Staging and Prognostic Stratification in Anal Canal Cancer
Source: Cancers (Basel). 2025 Nov 14;17(22):3653. doi: 10.3390/cancers17223653 (PMC12651940; doi:10.3390/cancers17223653)
Supplement: Supplementary file 1 [file cancers-17-03653-s001.zip › cancers-3919665-supplementary.pdf]

SUPPLEMENTARY TABLES

Supplementary Table S1. Univariate hazard ratios (HR) associated with progression from separate Cox PH models. Note that the HR are dependent on the distribution of the features and not readily interpretable.

COVARIATE	HR(95%CI)	P-VALUE
40%: SHAPE_Surface(mm2)[onlyFor3DROI] (log)	2.27 (1.68, 3.06)	<0.001
40%: GLRLM_GLNU (log)	1.98 (1.53, 2.55)	<0.001
40%: CONVENTIONAL_SUVbwcalciumAgatstonScore[onlyForCT] (log)	1.86 (1.46, 2.37)	<0.001
40%: SHAPE_Volume(mL) (log)	1.85 (1.46, 2.36)	<0.001
40%: SHAPE_Volume(vx) (log)	1.85 (1.45, 2.36)	<0.001
40%: GLZLM_GLNU (log)	2.02 (1.51, 2.70)	<0.001
40%: GLRLM_RLNU (log)	1.78 (1.39, 2.29)	<0.001
40%: NGLDM_Coarseness (log)	0.51 (0.38, 0.69)	<0.001
40%: CONVENTIONAL_TLG(mL)[onlyForPETorNM] (log)	1.63 (1.29, 2.06)	<0.001
40%: DISCRETIZED_TLG(mL)[onlyForPETorNM] (log)	1.63 (1.29, 2.05)	<0.001
40%: NGLDM_Busyness (log)	1.89 (1.34, 2.66)	0.002
40%: SHAPE_Compacity[onlyFor3DROI] (log)	4.44 (1.69,11.71)	0.018
40%: GLZLM_ZLNU (log)	1.41 (1.07, 1.87)	0.092
40%: GLZLM_ZP	0.14 (0.03, 0.73)	0.096
40%: GLCM_Contrast[=Variance] (log)	0.73 (0.54, 0.97)	0.12
40%: GLCM_Dissimilarity (log)	0.57 (0.33, 1.00)	0.12
40%: GLZLM_SZE	0.16 (0.03, 0.83)	0.12
40%: GLCM_Homogeneity[=InverseDifference] (log)	1.98 (0.81, 4.84)	0.13

HR = hazard ratio; CI = confidence interval; SUVbw = standardized uptake value normalized by body weight; TLG = total lesion glycolysis; 3DROI = three-dimensional region of interest; GLCM = gray level co-occurrence matrix; GLRLM = gray level run length matrix; GLNU = gray level nonuniformity; RLNU = run length nonuniformity; NGLDM = neighboring gray level dependence matrix; GLZLM = gray level zone length matrix; ZLNU = zone length nonuniformity; ZP = zone percentage; SZE = small zone emphasis. All radiomic features were extracted using a 40% SUV threshold, and hazard ratios were calculated using log-transformed feature values.

Supplementary Table S2. PET radiomic features that discriminate between patients with stage III/IV vs stage I/II disease based on PET AJCC v9. Wilcoxon rank sum test was used to compare

the difference in distributions between groups, Holm's corrections were made to the p-values to control for multiple comparisons and features with adjusted p-values <0.05 are shown.

COVARIATE	I/II (N=44)	III/IV (N=79)	STAGE DIFFERENCE P-VALUE
40%: CONVENTIONAL_SUVbwcalciumAgatstonScore[onlyForCT] (log)	9.0 (8.4-9.5)	10.3 (9.9-11.0)	<0.001
40%: CONVENTIONAL_TLG(mL)[onlyForPETorNM] (log)	4.1 (3.3-4.5)	5.4 (4.9-6.2)	<0.001
40%: DISCRETIZED_TLG(mL)[onlyForPETorNM] (log)	5.2 (4.5-5.7)	6.6 (6.1-7.4)	<0.001
40%: SHAPE_Volume(mL) (log)	1.8 (1.2-2.2)	3.1 (2.7-3.8)	<0.001
40%: SHAPE_Volume(vx) (log)	5.7 (5.1-6.1)	7.0 (6.6-7.6)	<0.001
40%: SHAPE_Surface(mm2)[onlyFor3DROI] (log)	7.6 (7.2-7.9)	8.7 (8.3-9.2)	<0.001
40%: SHAPE_Compacity[onlyFor3DROI] (log)	1.1 (0.9-1.2)	1.3 (1.1-1.6)	<0.001
40%: GLRLM_GLNU (log)	2.8 (2.3-3.4)	4.0 (3.5-4.4)	<0.001
40%: GLRLM_RLNU (log)	5.4 (5.0-5.9)	6.8 (6.2-7.3)	<0.001
40%: NGLDM_Coarseness (log)	-4.2 (-4.5-(-3.8)	-5.3 (-5.7-(-4.8)	<0.001
40%: NGLDM_Busyness (log)	-1.8 (-2.4-(-1.2)	-1.2 (-1.6-(-0.7)	0.001
40%: GLZLM_GLNU (log)	2.1 (1.7-2.4)	3.2 (2.7-3.7)	<0.001
40%: GLZLM_ZLNU (log)	4.3 (3.5-4.7)	5.4 (4.9-5.8)	<0.001

SUVbw = standardized uptake value normalized by body weight; TLG = total lesion glycolysis; 3DROI = three-dimensional region of interest; GLRLM = gray level run length matrix; GLNU = gray level nonuniformity; RLNU = run length nonuniformity; NGLDM = neighboring gray level dependence matrix; GLZLM = gray level zone length matrix; ZLNU = zone length nonuniformity. Values in parentheses represent interquartile ranges. All radiomic features were extracted using a 40% SUV threshold.
